# Lightweight, Fiber-Damage-Resistant, and Healable Bio-Inspired Glass-Fiber Reinforced Polymer Laminate

**DOI:** 10.3390/polym14030475

**Published:** 2022-01-25

**Authors:** Jia Long Liu, Lorenzo Mencattelli, Jie Zhi, Ping Yee Chua, Tong-Earn Tay, Vincent Beng Chye Tan

**Affiliations:** 1 Department of Mechanical Engineering, National University of Singapore, 9 Engineering Drive 1, Singapore 117565, Singapore; e0011565@u.nus.edu (J.L.L.); jie.zhi@nus.edu.sg (J.Z.); e0176357@u.nus.edu (P.Y.C.); mpetayte@nus.edu.sg (T.-E.T.); 2 Helicoid Industries Inc., 82663 Redford Way, Indio, CA 92201, USA; lorenzom@helicoidind.com

**Keywords:** lightweight, bio-inspired helicoidal structure, composite healing, impact, glass-fiber-reinforced polymer

## Abstract

Glass-Fiber-Reinforced Polymer (GFRP) laminates are widely used in the automotive and marine industries such as auto bodies and boat hulls. Decreasing the weight and improving the reparability of GFRP parts will cut down material usage, fuel consumption and repair costs. This study shows a bio-inspired helicoidal stacking configuration that significantly improves the impact performance and fiber damage resistance of GFRP laminates. For similar impact performance in terms of perforation energy, the helicoidal GFRP laminate is 20% lighter than the conventional quasi-isotropic GFRP laminate. Upon impact, delaminations and matrix splits link-up and grow extensively throughout the helicoidal laminate. This effectively reduces fiber damage and improves impact performance. Because helicoidal GFRP laminates are resistant to fiber damage and composite healing agents can effectively repair non-fiber damage, embedding healing agents into helicoidal GFRP results in lightweight, inexpensive and healable laminates.

## 1. Introduction

Glass-Fiber-Reinforced Polymer (GFRP) laminates have been widely adopted in the automotive and marine industries due to their low cost and good mechanical performance [1,2,3]. Since auto bodies and boat hulls require a large quantity of GFRP laminates, decreasing the weight of GFRP parts while maintaining their mechanical performance is beneficial for the industry and environment as it saves material costs and cuts down fuel consumption while meeting safety requirements. Improving the repair efficiency (cost/time ratio) of GFRP parts is also beneficial because damaged composite parts are expensive and difficult to repair [4].

Many researchers have attempted to strengthen and toughen GFRP parts by adding micro/nano-reinforcements [5,6,7,8] or by improving manufacturing processes [9]. However, many of these approaches increase either the material and/or fabrication cost. Since many high-volume composite components make extensive use of lower strength, lower cost GFRP laminates instead of higher performance and more costly Carbon-Fiber-Reinforced Polymer (CFRP) laminates, it is essential to develop design strategies to increase damage resistance, reduce weight, reduce material usage and reduce maintenance costs with GFRPs.

Drawing inspiration from nature, it is proposed that the mechanical performance of GFRP laminates can be improved through bio-inspired structural design. Many crustaceans’ exoskeletons are composed of layers of aligned chitin fibers in a similar fashion to manmade fiber-reinforced polymer laminates [10]. The weak chitin fibers have to provide protection against out-of-plane loading and impact; at the same time, the exoskeleton also needs to be thin and light so as to not impair mobility nor increase energy consumption—a similar requirement for GFRP parts. Weaver et al. [10] investigated the microstructure of crustacean exoskeletons and found various mechanisms working in synergy to improve impact damage resistance. Most notable was the helicoidal structure of laminated chitin fibers [11].

In the helicoidal structure, the orientation of the fibers in one layer is offset at a slight angle with respect to the fibers in the layer below, forming a spiraling helicoidal configuration. Inspired by these findings, several researchers have conducted studies that mimic the helicoidal structure using CFRP laminates [12,13,14,15,16,17,18,19,20,21,22,23,24,25,26]. The results show that helicoidal layup significantly improves low-speed impact [12,13,14,15,16,17] and high-speed impact performance [18] of CFRP laminates. Furthermore, a recent study shows that the helicoidal structure also improved the high-speed impact perforation energy of natural fiber (flax)-reinforced polymer composites by 30% [20]. Since the strength and stiffness of glass fibers lie between carbon fiber and flax fiber, it is reasonable to expect that a helicoidal layup would also improve the impact performance of GFRP laminates. Many GFRP parts such as the roof of automobiles and boat hulls mainly provide protection against out-of-plane impact while the structural supports of these parts are provided by other components such as beams and trusses. Therefore, lightweight helicoidal GFRP laminates can offer an effective solution to enhance out-of-plane impact resistance and decrease structural weight.

Several studies [18,20,22,23,24,25,26] reported that the helicoidal structure also reduces fiber damage in CFRP laminates as well as natural-fiber-reinforced polymer laminates. This is achieved by dissipating energy through mainly helicoidal matrix damage, namely, diffused matrix cracks and delaminations. With regard to conventional composites with high-performance fibers such as carbon and glass, matrix damage can be healed if a re-processable matrix is used (e.g., thermoplastic) or if healing agents are dispersed within the matrix [26]; however, fiber damage is irreversible. Therefore, helicoidal GFRP laminates with an embedded healing agent may also possess good healing properties.

This study investigates the impact performance of helicoidal GFRP laminates together with conventional quasi-isotropic GFRP laminate as reference. Helicoidal GFRP laminates with fewer plies were fabricated to determine the percentage weight reduction achieved while maintaining similar impact performance as the reference laminate. A lightweight and healable helicoidal GFRP laminate was produced from investigating the damage patterns to understand the cause of failure.

## 2. Experimental Setup

### 2.1. Fabrication Processes

Quasi-isotropic and helicoidal laminates were fabricated from unidirectional G10000/6510 Glass fiber-epoxy prepregs. The mechanical properties of the material are shown in Table 1. Individual prepregs were cut into 100 × 100 mm^2^ squares. They were then laid manually to form 61-ply quasi-isotropic and helicoidal laminates, as well as lighter 49-ply and 46-ply helicoidal configurations, as shown in Table 2.

**Figure 1:**
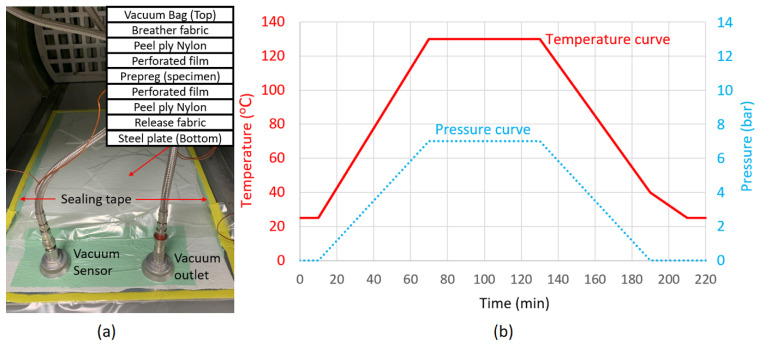
Laminate curing setup (**a**) and curing cycle (**b**).

The specimens were cured in an autoclave. The layups and curing cycle are shown in Figure 1. The temperature was raised from room temperature to 130 °C over 1 h and maintained for 1 h. The specimens were then cooled over 1.5 h to room temperature resulting with an average laminate thickness of 4.5 ± 0.05 mm for QI61 and HL61, 3.6 ± 0.05 mm for HL49 and 3.4 ± 0.05 mm for HL46.

### 2.2. Perforation IMPACT Test

**Figure 2:**
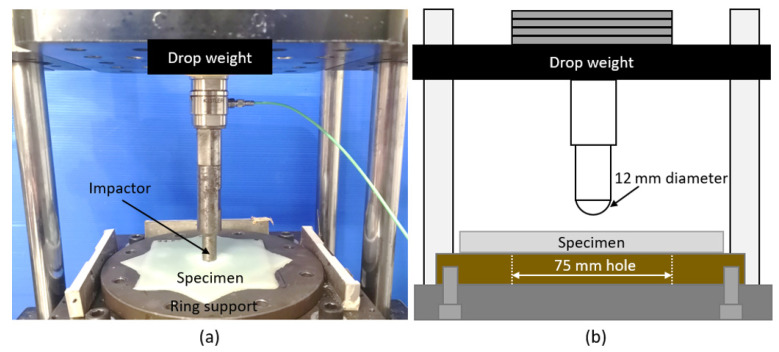
Drop test experimental setup (**a**) and schematic diagram of the test (**b**).

Out-of-plane impact tests were conducted to evaluate the energy required to perforate the specimens. As shown in Figure 2, the specimens were placed on a plate with a 75 mm circular hole, which allows the specimens to flex under impact while providing an out-of-plane constraint that is independent of in-plane orientation. The specimens were impacted by a 12 mm diameter hemispherical impactor attached to a drop weight. The drop weight was set to drop from 50 cm height to achieve an impact velocity of 3.1 m/s. The drop weight mass can be increased or decreased, and the impact energy is adjustable with an interval of 1 J. The perforation energy is taken as the lowest energy at which the test specimen is perforated.

### 2.3. Fabrication Process of Healable Helicoidal GFRP Laminate

Additional light weight helicoidal laminates that match the perforation energy of QI61 were fabricated and cured using the same process as described in Section 2.1, except with an additional healing agent (0.95% weight fraction, Nylon6 healing agent [26,28]).

**Figure 3:**
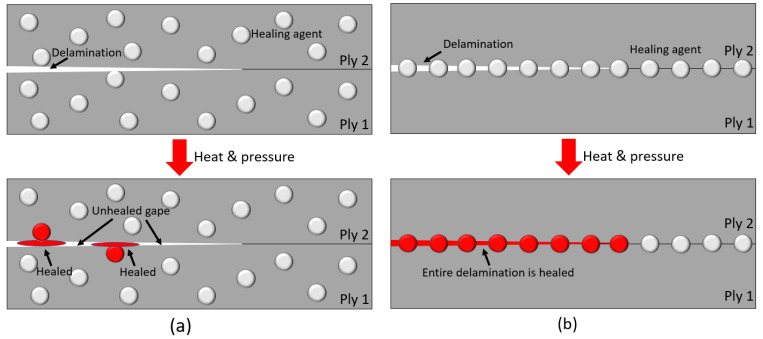
Dispersed healing agent within plies (**a**), healing agent between plies (**b**).

**Figure 4:**
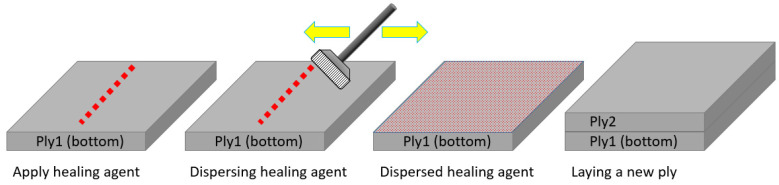
Schematic diagram of the healable laminate fabrication process.

Dispersing healing agents within plies is commonly adopted for composite healing [29,30,31,32,33]. The healing agent is in a form of micro-capsules (self-healing healing agent) or powder (thermoplastic healing agent) and is mixed with resin and dispersed into each ply, as shown in Figure 3a. Thermoplastic healing agents melt under heat and flow into nearby cracks under pressure, healing the epoxy cracks, as shown in Figure 3a. However, the melted healing agents may not fully spread across the entire delaminated interface because most healing agents are dispersed within the plies. Since delamination is a dominant damage in helicoidal laminates [12,22], it is more effective to apply healing agents between plies as shown in Figure 3b. Therefore, healable specimens were produced with a Nylon6 healing agent added between plies, as shown in Figure 4.

### 2.4. Impact and Healing of Helicoidal GFRP Laminate

The healable specimens were then subjected to a low energy impact. After impact, the damaged specimens were healed in a hot-press. The specimen was firstly heated to 200 °C over 10 min to soften the healing agent before a pressure of 2 bar was applied. The specimen was then heated to 220 °C over 20 min to allow the Nylon6 healing agent to melt and flow into the cracks. The specimen was then cooled to room temperature over 15 min.

## 3. Results and Discussion

**Figure 5:**
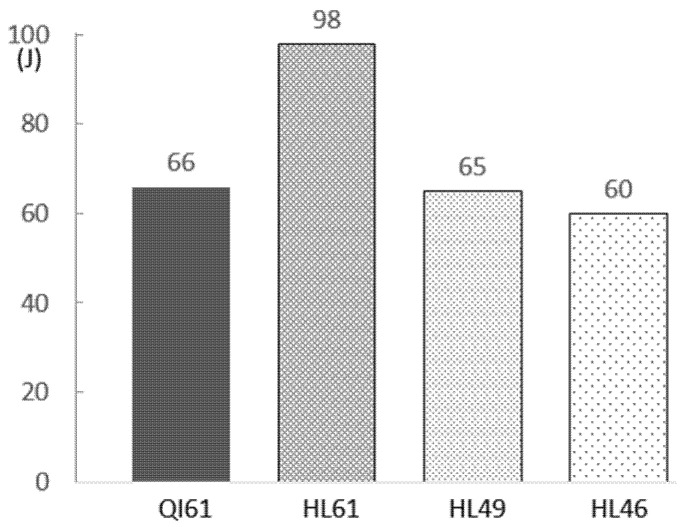
Perforation energy of the specimens.

As shown in Table 3 and Figure 5, the perforation energy of HL61 is 48% higher than QI61. This shows that the impact performance of GFRP laminates can be significantly improved by stacking the laminate helicoidally. The perforation energy of HL49 is 1 J lower than QI61, but it is 20% lighter in weight. The perforation energy of HL46 is 6 J lower than QI61. Since HL49 provided the closest value of perforation energy compared to QI61, additional QI61, HL61 and HL49 were subjected to a 16 J impact to inspect their damage pattern under lower energy impacts.

**Figure 6:**
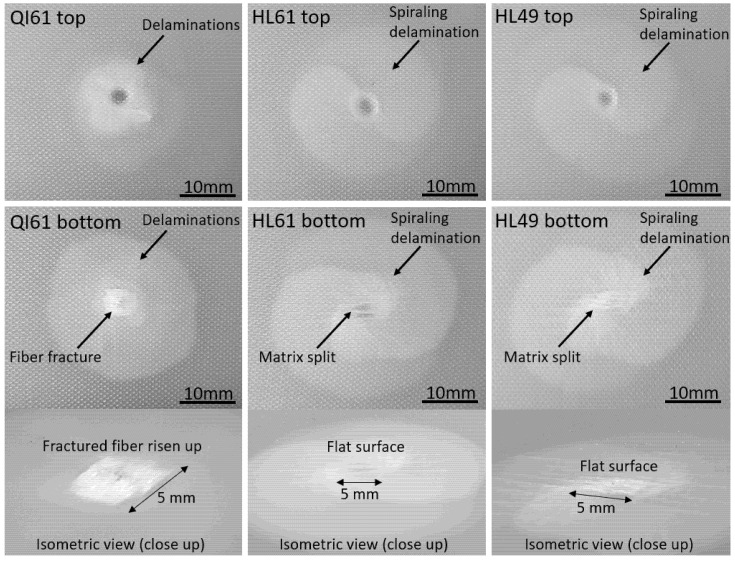
Damage to the specimens after low energy (16 J) impact.

**Figure 7:**
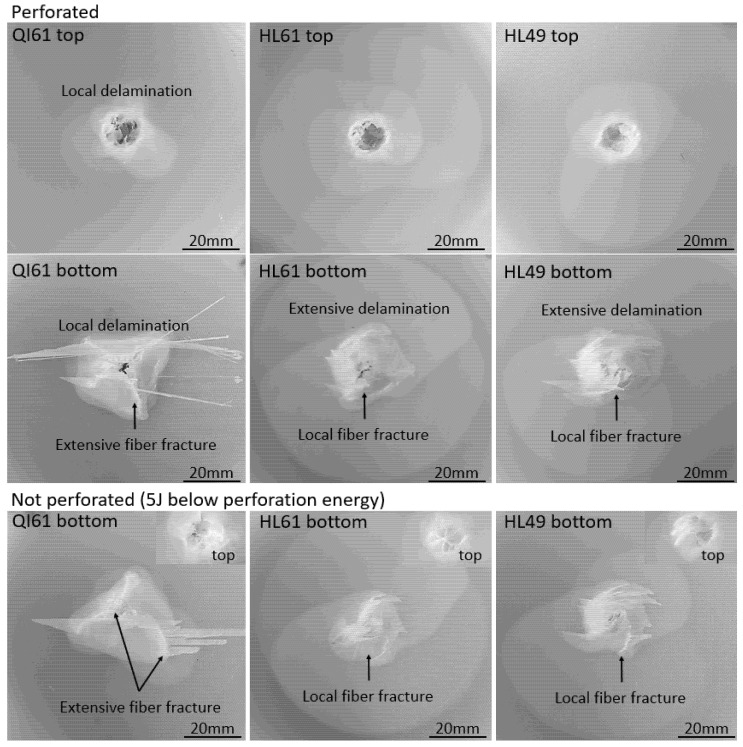
Damage to the perforated and nearly perforated samples.

As shown in Figure 6 and Figure 7, the helicoidal GFRP laminates clearly show highly diffused spiraling delamination damage. The extent of delamination is small near the bottom of the laminate but increases dramatically from the bottom towards the middle of the laminate, and it then decreases near the top of the laminate. The delamination in each interface rotates by a small angle from the previous one, and these delaminations are linked by matrix splits and spiral upwards, similar to a pair of spiral staircases. This is the unique damage pattern of helicoidally stacked fiber-reinforced laminates and is referred to as a spiraling matrix split [18,22,25]. Liu et al. [25] and Suksangpanya et al. [34] reported that spiraling matrix split is a form of matrix crack that grows between fibers. The resistance to the propagation of a spiraling matrix split in a helicoidal laminate decreases as the inter-ply angle of the laminate decreases [34], and the spiraling matrix split propagates extensively in helicoidally stacked laminates with small inter-ply angles, such as in the cases of HL61 and HL49.

Although spiraling matrix split was deduced through theoretical study and experimental CT-scan by Suksangpanya et al. [34] and Liu et al. [22], respectively. Figure 6 and Figure 7 show, for the first time, the images of the spiraling matrix split directly observable from the surface of the composite. This was possible due to the semitransparency of GFRP laminates. The pair of spiraling matrix splits in HL61 are almost identical, whereas one of the spiraling matrix splits in HL49 was more extensive when the impact energy reached perforation energy. This is mainly related to the fact that the HL61 has a more balanced 4 revolutions (1 revolution is 180°) from 0° to 720° while HL49 has an unbalanced 3 revolutions from 0° to 540°, so the spiraling matrix split tends to develop unevenly in HL49 when the impact energy is large.

As shown in Figure 6, under the same 16 J low energy impact, fiber damage is developed at the bottom of QI61. Despite more extensive matrix cracks, no fiber damage is observed at the bottom surface of HL61 and HL49. As shown in Figure 7, fiber damage extends beyond the point of impact in perforated QI61, whereas fiber damage is contained within a narrower region in perforated HL61 and HL49. When impact energy is 5 J lower than the perforation energy of each laminate, extensive fiber damage is again observed at the bottom of QI61 while minimal fiber damage is observed at the bottom of HL61 and HL49. On the other hand, delamination in QI61 is localized compared to the size of the spiraling matrix split in HL61 and HL49.

The reduction in fiber damage in helicoidal laminates is a consequence of the extensive sub-critical spiraling matrix split. The highly diffused combination of matrix splits and delaminations in the spiraling matrix split reduces stresses along the fiber direction which minimizes fiber damage [35,36,37,38]. As a result, under different impact intensities, fiber damage is the dominant damage in quasi-isotropic GFRP laminates whilst both spiraling matrix split and fiber damage are prevalent in helicoidal GFRP laminates.

Perforation of GFRP laminates is the result of an impactor breaking through the fibers along its path. Since helicoidal GFRP laminates are more resistant to fiber damage and the impact energy is dissipated through fiber damage as well as the massive spiraling matrix split, this is the reason why helicoidal GFRP laminate excels in impact performance.

Several studies on composite intrinsic healing technology have shown that damaged epoxy resin can be healed effectively with thermoplastic healing agents [30,31,32,33]. On the other hand, damaged fibers in the composite will not recover their full strength after healing because the healing agent is weaker than the fibers. The healing agent simply bonds the fractured fibers and leaves a weak spot. Since fiber damage is diverted to delamination and matrix damage during impact for helicoidal GFRP laminates, helicoidal GFRP laminates with embedded healing agents would facilitate good healing.

**Figure 8:**
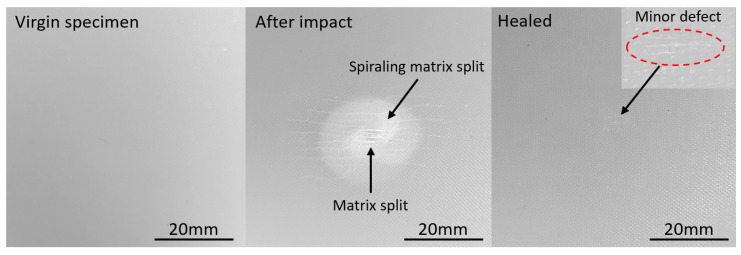
Bottom surface of healable HL49 specimen after impact and after healing.

HL49 specimens with embedded Nylon6 healing agent following the fabrication process described in Section 2.3 were produced. In addition, they were then subjected to 16 J impact and healed following the process described in Section 2.4. As shown in Figure 8, the damage within the healable HL49 after impact is similar to the HL49 without healing agents shown in Figure 6. The spiraling matrix split is developed near the point of impact with an extensive matrix split at the bottom surface of the laminate. After the healing process, most of the cracks have closed up except for a few superficial cracks at the bottom of the laminate.

The result shows that by adding a small amount of healing agent between each ply, the GFRP helicoidal laminates can achieve good healing after low energy impact.

## 4. Conclusions

This study shows that by stacking GFRP laminate helicoidally, the weight of the laminates can be reduced by 20% while maintaining the same perforation energy as the conventional quasi-isotropic GFRP laminate under impact. Since a large quantity of GFRP laminates are used in the automotive and marine industry as auto body parts and boat hulls, and since the lighter helicoidal GFRP laminates can meet the same safety requirement as the conventional GFRP laminate, adopting helicoidal GFRP laminates is beneficial for the industry and the environment as it saves material and cuts down fuel consumption while not introducing additional costs.

During impact, a highly diffused spiraling matrix split develops in helicoidal GFRP laminates, reducing fiber damage. In contrast, damage is highly localized in conventional GFRP quasi-isotropic laminates, leading to localized delaminations and extensive fiber damage. Helicoidal GFRP laminates are more resistant to perforation because of the better resistance to fiber damage and the development of diffused spiraling matrix splits. Helicoidal GFRP laminates thus show reduced fiber damage under low energy impact. As such, healable helicoidal GFRP laminate can be produced by simply adding thermoplastic healing agents between plies.

## Figures and Tables

**Table 1:** Mechanical and physical properties of G10000/6510 Glass fiber–epoxy prepreg.

Property	Value	Remarks
Ply thickness	0.074 mm	
Ply areal density	0.1475 kg/m^2^	
Modulus (fiber direction), E_1_	61.6 GPa	ASTM D3039
Modulus (Transverse direction), E_2_	11.8 Gpa	ASTM D3039
Tensile strength (fiber direction), σ_1t_	1146 Mpa	ASTM D3039
Compressive strength (fiber direction), σ_1c_	597 Mpa	ASTM D6641
Tensile strength (transverse direction), σ_2t_	38.5 Mpa	ASTM D3039
Compressive strength (transverse direction), σ_2c_	110 Mpa	ASTM D6641
Interlaminar Mode I fracture toughness	0.440 mJ/mm^2^	ASTM D5528
Interlaminar Mode II fracture toughness	1.12 mJ/mm^2^	ASTM D7905/D7905M
Intralaminar Mode I fracture toughness (transverse direction),	0.751 mJ/mm^2^	[27]
Inerlaminar shear strength	69.5 Mpa	ASTM D2344

**Table 2:** Laminate configurations.

Designation	Description	Laminate Area Density	Laminate Thickness	Weight Against QI61	Configuration
QI61	61 ply quasi-isotropic	9 kg/m^2^	4.5 mm	-	(0/90/45/−45)_7_/0/90/0/90/0/(−45/45/90/0)_7_
HL61	61 ply helicoidal	9 kg/m^2^	4.5 mm	100%	0/−12/−24/−36…/−720
HL49	49 ply helicoidal	7.2 kg/m^2^	3.6 mm	80.3%	0/−11.25/−22.5/−33.75…/−540
HL46	46 ply helicoidal	6.8 kg/m^2^	3.4 mm	75.4%	0/−12/−24/−36…/−540

**Table 3:** Perforation impact test results.

Designation	Specimen No.	Impact Energy (J)	Perforation
QI61	1	50	No
	2	60	No
	3	70	Yes
	4	65	No
	5	68	Yes
	6	66	No
	7	67	Yes
	8	66	Yes
	9	61	No
HL61	1	80	No
	2	100	Yes
	3	97	No
	4	98	No
	5	99	Yes
	6	98	Yes
	7	94	No
HL49	1	66	Yes
	2	60	No
	3	65	No
	4	64	No
	5	66	Yes
	6	65	No
HL46	1	60	No
	2	63	Yes
	3	61	Yes
	4	60	Yes
	5	59	No
	6	55	No

## Data Availability

The data presented in this study are available on request from the corresponding author.
